# Fresh Vaccine Lymph

**Published:** 1844-11

**Authors:** 


*Fresh Vaccine Lymph.*—M. Majendie announced to the Academy of Sciences, May 27th, that he had met with the cow-pox in a cow which belonged to him. Dr. Fiard has vaccinated many infants with it, and genuine vaccine pustules were developed. M. Majendie placed the cow at the disposal of the Academy.—*Ibid.*

